# Prevalence and prognosis of organ dysfunctions of AIDS critically ill patients

**DOI:** 10.1186/cc12405

**Published:** 2013-03-19

**Authors:** AJ Japiassu, RA Amâncio, D Medeiros, EM Mesquita, EM Motta, FA Bozza

**Affiliations:** 1 Oswaldo Cruz Foundation, Rio de Janeiro, Brazil

## Introduction

The aim was to analyze the prognosis of AIDS patients with organ dysfunctions at ICU admission.

## Methods

A prospective cohort study, including all patients with HIV/ AIDS diagnosis, who were admitted to a specialized ICU from November 2009 until May 2012. Patients with less than 24 hours of ICU stay were excluded. Demographics and nutritional status were collected. The organ dysfunctions were classified according to the SOFA score, and categorized as absent (0 SOFA point), mild (1 to 2 points) and severe (3 to 4 points). We expressed numeric variables as median and interquartile interval (25% to 75%). We performed a multivariate analysis of possible variables associated with hospital mortality (*P *0.2), and we explored the 7-day, 28-day and 60-day survival of patients with and without independent risk factors.

## Results

We included 139 patients with HIV/AIDS admitted to the ICU. Median age was 40 (31 to 48) years and 71% were male. Severe malnutrition was common (34%). The CD4 cell count was 84 (25 to 274) cells/mm^3 ^and viral load was 17,733 (67 to 174,214) copies/ml; 57% had at least one opportunistic infection; 55% had used antiretroviral therapy previous to ICU admission. Mechanical ventilation was used by 46% of patients and hospital mortality was 42%. Total SOFA score was 5 (2 to 9) points. Cardiovascular dysfunction was the most common on the first days of stay (51%), followed by respiratory (42%), neurological (40%), renal (35%), hematological (27%) and hepatic (17%). Cardiovascular and renal dysfunctions presented with higher rate of severe dysfunction (30% and 15%, respectively). Rates of neurological (*P *= 0.002), renal (*P *= 0.009) and hematological (*P *= 0.003) dysfunctions were higher in nonsurvivors. Age, CD4 cell count, malnutrition, and opportunistic infections were included in the multivariate analysis. Neurological dysfunction was the independent risk factor for hospital mortality (odds 3.2 (1.4 to 7.2)). The presence of neurological dysfunction was dichotomized: associated or not with primary neurological diagnosis; survival was lower in the patients with neurological dysfunction and without primary neurological diagnosis (log-rank test 0.001 in the 7-day and 0.02 in the 28-day analysis). Sixty-day survival was similar in primary and secondary neurological dysfunction, but it remained lower than in patients without neurological impairment.

## Conclusion

Neurological dysfunction was independently associated with hospital survival, mainly in those AIDS critically patients without primary neuropathy.

